# Strategic testing following toxic epidermal necrolysis allows reintroduction of chemotherapy in a patient with progressive myeloma

**DOI:** 10.5415/apallergy.0000000000000125

**Published:** 2023-12-18

**Authors:** Daniel Soon Lee Goh, Ramon Yuson, Praveen Gounder, James Yun, Sandhya Limaye

**Affiliations:** 1 Department of Immunology, Concord Hospital, Sydney, Australia; 2 Department of Haematology, Concord Hospital, Sydney, Australia; 3 Department of Immunology, Prince of Wales Hospital, Sydney, Australia; 4 University of Sydney, Infectious Diseases and Immunology, Darlington, New South Wales, Australia

**Keywords:** ALDEN, chemotherapy, patch test, toxic epidermal necrolysis, severe cutaneous adverse reaction

## Abstract

Toxic epidermal necrolysis (TEN) and Stevens–Johnson syndrome belong to a family of severe cutaneous adverse reactions that can be life-threatening and carry a risk of significant morbidity and potential mortality in the event of re-exposure. Lifelong avoidance of the culprit agent is mandated, which can lead to the exclusion of multiple medications if the trigger is unclear. This can result in adverse health outcomes analogous to that of a penicillin allergy label. We present a case in which the patient would progress to fatal myeloma in the absence of treatment, however, multiple medications were administered prior to the occurrence of TEN following previous chemotherapy. Available risk stratification tools including human leucocyte antigen assessment and the algorithm of drug causality for epidermal necrolysis scoring system were utilized followed by patch testing which identified a lesser-suspected agent as possibly causative. Further evidence-based in vivo testing and subsequent challenges allowed for the reintroduction of life-saving chemotherapy.

## 1. Introduction

Stevens–Johnson syndrome (SJS) and toxic epidermal necrolysis (TEN) are life-threatening severe cutaneous adverse drug reactions (SCAR) that mandate lifelong avoidance of the causative agent. Definitive identification of the latter can be difficult in the context of inadequate history or multiple drug exposure, which can lead to the exclusion of multiple medications and restriction of subsequent therapeutic options. This is further compounded by limited risk-free testing options. Diagnostic in vivo tests are relatively contraindicated, given the significant morbidity and potential mortality associated with drug re-exposure [1]. We present a safe and strategic diagnostic approach to assessment of drug causality in a patient who developed TEN following exposure to combination chemotherapy, that allowed successful reintroduction of life-saving treatment.

## 2. Case report

A 73-year-old male was referred with a history of TEN following exposure to several medications for treatment of multiple myeloma. TEN occurred after his second cycle of chemotherapy and required treatment with intravenous immunoglobulin and prolonged intensive care and specialist burns admission for severe disease with 70% body surface area involvement and a score of TEN severity (SCORTEN) score of 4. Antecedent drugs were allopurinol, trimethoprim/sulfamethoxazole, bortezomib, lenalidomide, dexamethasone, valaciclovir, and pantoprazole. He recovered from TEN, but was advised to avoid all possible culprits, thus treatment for multiple myeloma was deferred. Months later, a progressive rise in paraprotein level and progressive anemia warranted retreatment of myeloma. Preferred second-line treatment was with daratumumab, bortezomib and dexamethasone (DVd); carfilzomib with dexamethasone was considered a less favored but acceptable alternative.

We agree that best practice following TEN dictates lifelong avoidance of implicated agents, however, in this case, progressive myeloma would be ultimately life-limiting, together with limited plausibility that all antecedent medications were causative. We first calculated algorithm of drug causality for epidermal necrolysis (ALDEN) scores for all possible culprit medications (Table 1) and assessed human leucocyte antigen (HLA)-B*58:01 status due to allopurinol exposure.

**Table 1.** ALDEN scores of medications administered prior to toxic epidermal necrolysis reaction

Medication	ALDEN score
Allopurinol	6
Sulfamethoxazole	6
Lenalidomide	3
Dexamethasone	3
Bortezomib	2
Valacyclovir	2
Pantoprazole	2

ALDEN, algorithm of drug causality for epidermal necrolysis.

**Figure 1.**
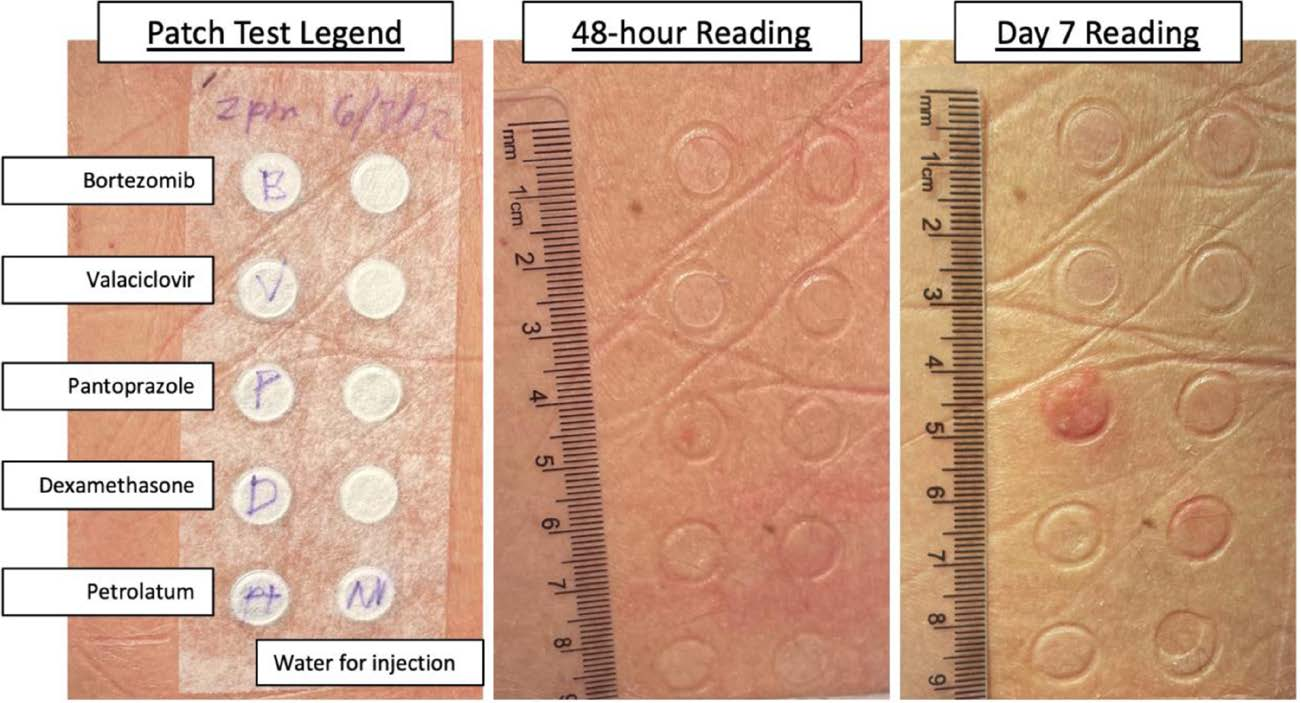
Patch testing results with readings at 48 hours and day 7. Pantoprazole demonstrates a positive patch test at day 7.

Detection of HLA-B*58:01 expression and ALDEN scores most strongly implicated allopurinol and sulfamethoxazole, necessitating lifelong avoidance of these agents. Extemporaneous patch tests were then performed to bortezomib, valaciclovir, pantoprazole, and dexamethasone using recommended published concentrations [2-4] (Fig. 1). A positive reaction was observed to pantoprazole, with erythema and papulation at 48 hours. All other patch tests were negative. Pantoprazole was eliminated from further investigations and lifelong avoidance recommended.

Skin prick and intradermal tests (SPT/IDT) were subsequently performed to both bortezomib (ALDEN score 2, negative patch test, and other likely causative drugs identified) and carfilzomib (no history of exposure, low predicted crossreactivity) as per a published protocol [2]. Both agents demonstrated mild erythema at 48 hours, more consistent with an irritant reaction rather than a true positive. SPT/IDT to dexamethasone (ALDEN score 3, negative patch test, other likely causative drugs identified) at recommended concentrations [5] were negative at 20 minutes and 48 hours.

The final stage of testing was performed prior to proposed myeloma treatment. Graded challenge to intravenous dexamethasone (1 mg day 0, 20 mg day 5) was administered without reaction. Finally, a graded challenge to bortezomib was undertaken, given negative patch test and SPT/IDT results. The patient received 0.2 mg subcutaneously (SC), followed by 2.4 mg SC 1 week later in the absence of a reaction to the test dose. Subsequent to the above, the patient has tolerated bortezomib, dexamethasone and daratumumab chemotherapy for myeloma without reaction. Notably, 2 of these agents had been administered prior to the development of TEN in previous treatment. Early reassessment following the commencement of DVd chemotherapy demonstrates a rapid response to treatment with a marked reduction in paraprotein concentration.

## 3. Discussion

Assessment of patients with a history of TEN is challenging, given its severity, frequent exposure to multiple possible culprit medications, and limited safe testing options. The correct identification of culprit medications in SCAR, particularly in SJS/TEN remains one of the biggest challenges in allergy, with no causal drug identified in one-third of cases [6]. When assessing a patient following TEN, the ALDEN scoring system allows assessment of likely drug causality and should be applied in the first instance [7]. Patch testing is increasingly recognized as a safe diagnostic tool in delayed drug hypersensitivity [8, 9], although yield is low and dependent upon a number of testing variables [9]. Furthermore, a negative patch test result does not exclude the drug as a cause of the reaction [9], hence the need to judiciously perform further diagnostic tests including IDT and ultimately challenges. A recent systematic review [10] demonstrates the utility of patch testing in TEN, with variable sensitivity for different drug classes, and high specificity. Reassuringly, only 3 of 82 patients experienced a systemic reaction following patch testing; all required topical corticosteroid management only and both cases of moderate severity occurred in patients with HIV infection and active tuberculosis. The authors suggest considerable caution in this setting. Finally, the finding of a positive patch test for pantoprazole is of interest in our case, given its identification as a potential agent of concern in the EuroSCAR study [11].

Currently available in research centers, the increasing breadth and availability of the lymphocyte transformation test and ELISpot assay [9] may be useful in the future. This case highlights an approach that utilizes available risk stratification tools alongside judicious, evidence-based cutaneous testing to investigate a patient with TEN following chemotherapy in the context of progressively worsening features of hematological malignancy. Although a definitive causative agent was not identified, the strategy allowed identification of drugs at lower risk that could be further investigated. An alternative treatment regimen was subsequently introduced and tolerated. Further developments in allergy testing for SCAR may pave the way for safer strategies and allow better identification of culprit medications in SJS/TEN.

## Conflicts of interest

The authors have no financial conflicts of interest.

## Authors contributions

Daniel Soon Lee Goh reviewed all case information, performed testing and wrote manuscript. Ramon Yuson performed testing and reviewed manuscript. Praveen Gounder reviewed case information and reviewed manuscript. James Yun collaborated regarding testing procedure and reviewed manuscript. Sandhya Limaye reviewed case information, performed testing and wrote/reviewed manuscript.
